# Supplementary material for: Content-rich biological network constructed by mining PubMed abstracts
Source: BMC Bioinformatics. 2004 Oct 8;5:147. doi: 10.1186/1471-2105-5-147 (PMC528731; doi:10.1186/1471-2105-5-147)
Supplement: Additional File 2 — The original results of the above study (non-essential files are deleted to keep the file size under the limit set by BMC bioinformatics). [file 1471-2105-5-147-S2.bz2 › chilibotAdditionalFile2/dip30/273ID1922387E1093/html/MAPK1_JUN.html]

 


 **MAPK1** and **JUN** 
  
Found 2098 abstracts in PubMed, retrieved 30.  
 

 What does Google say? 
 PDF only 
| .edu only 

---

**Interactive relationship** (e.g. stimulation, inhibition, etc)

**Inhibitory relationship**- SP600125, an inhibitor of c  **jun**  N terminal kinase, activates CREB by a p38 MAPK  [ **MAPK1** ]  mediated pathway.  Ref: 12878189 Biochem Biophys Res CommunBiochem Biophys Res Commun,
**Stimulatory relationship**- This selective MAPK  [ **MAPK1** ]  inhibition was associated with a reduction in AP 1  [ **JUN** ]  activity, with no affect on NF kappaB activity.  Ref: 12865664 Shock, 2003
**Neutral relationship**- Apoptosis signal regulating kinase 1 ASK1, a MAPK  [ **MAPK1** ]  kinase kinase MAPKKK protein, is known to activate both c  **Jun**  N terminal kinase JNK and p38 via MAPK  [ **MAPK1** ]  kinase MKK 4 7 and MKK3 6, respectively.  Ref: 12869527 J Biochem (Tokyo), 2003

**Non-interactive relationship** (e.g. studied together, co-existance, homology, etc.)

- Sorbitol resulted in activation of the extracellularly responsive kinase ERK  [ **MAPK1** ] , p54 c  **Jun**  N terminal kinase JNK and protein kinase B PKB.  Ref: 12881532 J Biol ChemJ Biol Chem, 2003
- Western blots for AP 1  [ **JUN** ]  and its signal mediators Erk  [ **MAPK1** ]  and JNK showed that expression of Fos and JNK were decreased by the addition of FZFAT at 300 microg ml.  Ref: 12856862 Am J Chin Med, 2003
- Apoptosis signal regulating kinase1 ASK1 is a ubiquitously expressed mitogen activated protein kinase kinase kinase MAPKKK that activates the SEK1 c  **Jun**  N terminal kinase JNK and MKK3 MKK6 p38 MAPK  [ **MAPK1** ]  signaling cascades.  Ref: 12878192 Biochem Biophys Res Commun, 2003
- We demonstrate currently that treatment of cultured UISO Mel 1 human melanoma cells with betulinic acid leads to the activation of p38 and stress activated protein kinase c  **Jun**  NH 2 terminal kinase widely accepted proapoptotic mitogen activated protein kinases MAPKs with no change in the phosphorylation of extracellular signal regulated kinases antiapoptotic MAPK  [ **MAPK1** ] .  Ref: 12855667 Clin Cancer Res, 2003
- The expression of mitogen activated protein MAP kinases, including extracellular signal regulated kinase ERK  [ **MAPK1** ] , c  **Jun**  NH 2 terminal protein kinase JNK, and p38, was analyzed in experimental autoimmune encephalomyelitis EAE in rats.Western blot analysis showed that the three MAP kinases phosphorylated ERK  [ **MAPK1** ]  p ERK  [ **MAPK1** ] , p JNK, and p p38 were increased significantly in the spinal cords of rats with EAE at the peak stage as compared with the levels in controls p< ; 0.05, and both p ERK  [ **MAPK1** ]  and p JNK declined slightly in the recovery stage of EAE.  Ref: 12864979 J Neuroimmunol, 2003
- 2 We examined the role of the mitogen activated protein kinase MAPK  [ **MAPK1** ]  c  **jun**  N terminal kinase JNK in TNFalpha and IL 1beta induced GM CSF, RANTES and IL 8 production in HASMC by using a novel specific inhibitor for JNK SP600125.  Ref: 12871843 Br J Pharmacol, 2003
- We identified that serum starvation and Dox reduced phosphorylation of mitogen activated protein kinase extracellular signal regulated kinase ERK  [ **MAPK1** ]  kinase MEK, protein kinase C PKC alpha beta and c  **Jun**  NH 2 terminal kinase.  Ref: 12883037 Mol Cancer Ther, 2003
- The recovery of aconitase activity also correlates with the activation of members of the MAPKs, p38, c  **Jun**  N terminal kinase JNK and ERK  [ **MAPK1** ] , and the activation p38 and JNK is attenuated by inhibition of guanylate cyclase.  Ref: 12865320 Endocrinology, 2003
- Such molecules include nuclear transcription factors e.g. GATA 4, c  **Jun** , protein kinases e.g. 90 kDa ribosomal protein S6 kinase, MAPK  [ **MAPK1** ]  activated protein kinase 2, and ion exchangers channels e.g. the Na H exchanger 1.  Ref: 12878473 J Mol Cell Cardiol, 2003
- While overexpression of wild type Ras enhanced CpG ODN induced extracellular signal regulated kinase ERK  [ **MAPK1** ] , c  **Jun**  NH 2 terminal kinase JNK and NF kappaB activation, overexpression of RasN17 inhibited CpG ODN induced ERK  [ **MAPK1** ] , JNK and NF kappaB activation.  Ref: 12867418 J Biol ChemJ Biol Chem, 2003
- The major groups of MAPKs found in cardiac tissue include the extracellular signal regulated kinases ERKs, the stress activated c  **Jun**  NH2 terminal kinases SAPK JNKs, p38 MAPK  [ **MAPK1** ] , and ERK5 big MAPK 1  [ **MAPK1** ]  BMK1.  Ref: 12841640 Mol Cell Biochem, 2003
- In this study, we investigated the role of c  **Jun**  NH2 terminal kinase JNK, a member of the mitogen activated protein kinase MAPK  [ **MAPK1** ]  family, in lipopolysaccharide LPS stimulated inducible nitric oxide synthase iNOS expression and nitric oxide NO production in J774 murine macrophages.  Ref: 12869635 Mol PharmacolMol Pharmacol, 2003
- The differential sensitivity of cells to edelfosine s cytostatic and cytotoxic effects has been attributed to edelfosine induced changes in the activities of many enzymes, including c  **Jun**  NH2 terminal kinase JNK, extracellular signal regulated kinases ERK  [ **MAPK1** ] , p38 kinase, and poly ADP ribose polymerase PARP.  Ref: 12851688 Int J Oncol, 2003
- These results suggest that induction of the MMP 13 gene by TNF alpha is mediated by ERK  [ **MAPK1** ] , p38, and JNK MAP kinases as well as AP 1  [ **JUN** ]  and NF kappaB transcription factors.  Ref: 12878172 Exp Cell Res, 2003
- In the present study we used immunoblotting techniques to examine the effects of acute and repeated restraint stress on the phosphorylation state of three MAPKs, the extracellular signal regulated kinase Erk1 2, c  **Jun**  N terminal kinase stress activated protein kinase JNK SAPK and p38 MAPK  [ **MAPK1** ] , in different brain regions.  Ref: 12850571 Brain Res, 2003
